# Systematic Review of Multidisciplinary Chronic Pain Treatment Facilities

**DOI:** 10.1155/2016/5960987

**Published:** 2016-03-29

**Authors:** Samantha R. Fashler, Lynn K. Cooper, Eric D. Oosenbrug, Lindsay C. Burns, Shima Razavi, Lauren Goldberg, Joel Katz

**Affiliations:** ^1^Department of Psychology, York University, Toronto, ON, Canada M3J 1P3; ^2^Canadian Pain Coalition, Oshawa, ON, Canada L1J 8P7

## Abstract

This study reviewed the published literature evaluating multidisciplinary chronic pain treatment facilities to provide an overview of their availability, caseload, wait times, and facility characteristics. A systematic literature review was conducted using PRISMA guidelines following a search of MEDLINE, PsycINFO, and CINAHL databases. Inclusion criteria stipulated that studies be original research, survey more than one pain treatment facility directly, and describe a range of available treatments. Fourteen articles satisfied inclusion criteria. Results showed little consistency in the research design used to describe pain treatment facilities. Availability of pain treatment facilities was scarce and the reported caseloads and wait times were generally high. A wide range of medical, physical, and psychological pain treatments were available. Most studies reported findings on the percentage of practitioners in different health care professions employed. Future studies should consider using more comprehensive search strategies to survey facilities, improving clarity on what is considered to be a pain treatment facility, and reporting on a consistent set of variables to provide a clear summary of the status of pain treatment facilities. This review highlights important information for policymakers on the scope, demand, and accessibility of pain treatment facilities.

## 1. Introduction

Chronic pain is a major global health problem that affects approximately 37% of individuals in developed countries [1]. In Canada, it is estimated that as many as 25% of adults have a chronic pain condition [2]. Chronic pain places a substantial burden on the individual and can contribute to disability [3], depression [4], anxiety [5], and lowered quality of life [6]. There are also significant societal costs associated with chronic pain. In the United States, chronic pain is estimated to cost between 560 and 635 billion dollars annually [7], while, in Canada, direct costs to the health care system are estimated to be over 6 billion dollars and indirect costs due to job loss and sick days are estimated to be over 37 billion dollars annually [8, 9].

The development and maintenance of chronic pain is complex, affected by biological, psychological, and social factors [5, 10]. Consequently, the most effective pain treatment strategies target a variety of factors simultaneously, an approach that is used in multidisciplinary pain treatment facilities [11–13]. The services and standards of multidisciplinary treatment vary considerably, which led the International Association for the Study of Pain (IASP) to release guidelines regarding the classification and ideal standards of pain services [14]. IASP defines a “multidisciplinary pain center” (a Level 1 facility) as being staffed by a variety of health care professions with expertise in pain management, including physicians, nurses, mental health professionals, and physical therapists. The team should work together and be able to effectively assess and treat any pain problem. Training opportunities, education, and research should be available at the pain center. A “multidisciplinary pain clinic” (a Level 2 facility) must uphold the same standards of a multidisciplinary pain center, although they may not have training and research opportunities. Further classification includes “pain syndrome programs” that provide multidisciplinary care for one type of pain and “single modality therapy programs” that provide one type of treatment. A “pain treatment facility” is a general term that encompasses all of the above definitions [15].

Despite being considered the highest standard of care for chronic pain, there is limited research regarding the availability and characteristics of pain treatment facilities globally. In order to provide a comprehensive overview of the published literature, the Canadian Pain Coalition (http://www.canadianpaincoalition.ca/) sponsored the present systematic review as the first phase of a “Report Card on Pain.” This initiative aims to identify areas of strength and weakness in pain treatment facilities worldwide to help inform policymakers on targeted funding strategies to better tackle the challenge of pain in Canada and globally. The objective of the present paper is to systematically review the published literature of surveys on multidisciplinary pain treatment facilities to provide an overview of their availability, caseload, and wait times, as well as the facility characteristics, including available treatments and employed pain professionals.

## 2. Methods

### 2.1. Search Strategy

The systematic review was conducted according to PRISMA guidelines [16]. The review protocol was developed by the authors prior to beginning the searches. Eligibility criteria required articles to meet four study characteristics: (1) studies must describe pain treatment facilities directly with questionnaires or other data collection methods (excluding qualitative studies); (2) studies must be original research articles (i.e., no editorials, reviews); (3) more than one multidisciplinary pain treatment facility must be surveyed; and (4) questionnaires must describe a range of pain treatments rather than focusing on one type or category of treatment (e.g., acupuncture). At this stage, no restrictions were placed on report characteristics (e.g., language), publication status, location of study, or date of publication. Additionally, studies were not required to be published by an academic journal and could be published by an established organization.

The search strategy included searching academic article databases, asking experts in the field, and reviewing reference lists of included articles. No language restrictions were made for the initial search. Database searches were conducted using MEDLINE (1946 to August 2014), PsycINFO (1912 to August 2014), and CINAHL (1979 to August 2014). The last search was completed on September 23, 2014. All authors developed the search protocol and one author (Joel Katz) conducted the searches. The authors of all included articles were contacted for the original questionnaire used in their study.

The following search terms were used: pain; pain clinic^*∗*^; pain service^*∗*^; pain cent^*∗*^; pain facilit^*∗*^; pain management clinic^*∗*^; pain management service^*∗*^; pain management cent^*∗*^; pain management facilit^*∗*^; pain treatment clinic^*∗*^; pain treatment service^*∗*^; pain treatment cent^*∗*^; pain treatment facilit^*∗*^; survey^*∗*^; program evaluation; program assessment; program outcome; questionnaire^*∗*^. See Appendices A and B for a complete description of the search strategy and search terms.

### 2.2. Data Collection

Study titles and abstracts were reviewed for inclusion by one author (Joel Katz) according to the eligibility criteria. Articles selected for full-text review were independently reviewed by two authors (Joel Katz and Samantha R. Fashler). Disagreements were discussed until a consensus could be reached.

Data was transposed into a data extraction spreadsheet collaboratively created by three authors. The spreadsheet was modified as needed to accommodate new information from studies as they were reviewed. One author entered the extracted data from the studies into the spreadsheet (Samantha R. Fashler) and one author checked the data that was entered (Eric D. Oosenbrug). Study authors were contacted to receive a copy of the survey used in the project. Six authors provided a copy of the survey, one author responded to say they did not have a copy of the survey, five authors did not respond, and one survey was available in the appendix of the published study. In one case, results on the same survey were reported in two studies [17, 18]. To avoid bias, no duplicate information was extracted and nonoverlapping content was included in the extraction spreadsheet.

All variables included in the data extraction spreadsheet related to the assessment and evaluation of pain treatment facilities. Specifically, information related to following domains was extracted: (1) study characteristics, including a definition of pain treatment facilities used, search strategy, location and date surveyed, questionnaire used, and the response rate; (2) access to pain treatment facilities, including the rate of new and follow-up consultations and wait times; (3) pain treatments available, including medical approaches, physical therapies, and psychological services; and (4) characteristics of pain treatment facilities, including the employed health care professionals, staff, space, and equipment.

Risk of bias was assessed with a tool developed by the Cochrane Collaboration [19]. It is a domain-based evaluation tool, assessing the following: selection bias, performance bias, detection bias, attrition bias, reporting bias, and other biases (not addressed with the former biases).

Summary measures included standardized differences in means. Data transformation was used to increase comparability across reported data: specific alterations to original data are described in their respective table. No additional analyses were undertaken.

## 3. Results

### 3.1. Study Selection

**Figure 1:**
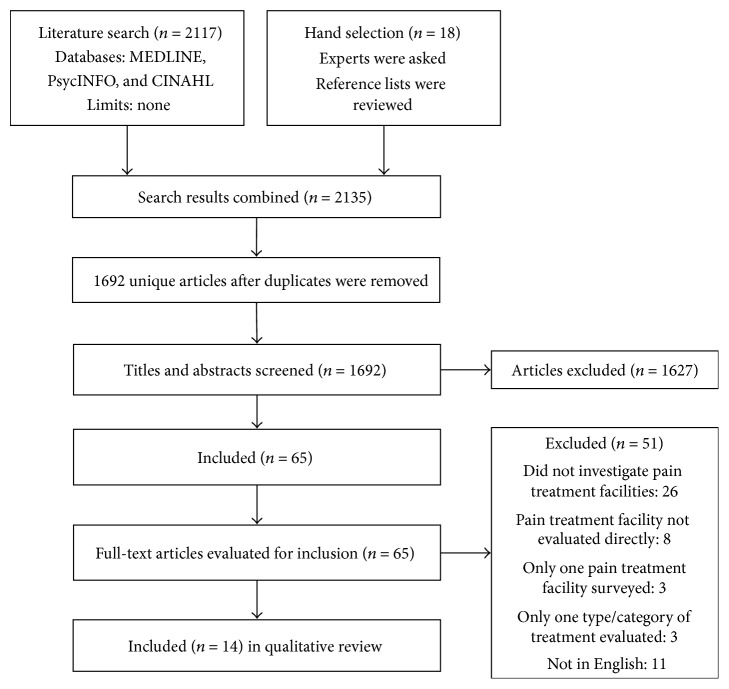
Flow diagram of study selection.

Searches conducted in MEDLINE, PsycINFO, and CINAHL yielded 2117 hits. Eighteen additional articles were hand selected by consulting with experts and searching reference lists. After duplicates were removed, 1692 unique articles remained. Articles were first screened by title and abstract by one author (Joel Katz), eliminating 1627 articles. The full texts of the remaining 65 articles were reviewed by two authors (Joel Katz and Samantha R. Fashler). Fifty-one articles were eliminated because they did not investigate pain treatment facilities (*n* = 26) or because they did not meet the selection criteria: eight articles surveyed clinicians or patients directly rather than pain treatment facilities, three only looked at one pain treatment facility, and three articles only examined one type of service. The original study design aimed to include non-English articles. However, the 11 non-English articles identified were eliminated from the present review due to difficulties in accurately translating them. See Figure 1 for a flow diagram of the articles selection and inclusion process used. In total, 14 studies were included in the present review.

Reporting bias may be present in the studies included in the review. When the questionnaires were available, the reported outcomes in the paper were compared to the questionnaire used to determine if any relevant data were not reported. The studies included in the review had a recruitment bias, since many pain treatment facilities that were contacted did not respond to the surveys, and are therefore not represented in the reported survey findings. A selection bias was present for the baseline characteristics of the surveys as the definition of a pain treatment facility was different for each study.

### 3.2. Study Characteristics

The 14 included studies were published between 1985 and 2013 [17, 18, 20–31]. Surveys were carried out around the world, with five in the United Kingdom, four in Canada, three in the United States, one in Australia, and one in Italy. Surveys reported countrywide or region-specific data. Ten were published in scientific journals [17, 18, 20–24, 29–31], two were audits of pain services [27, 28], and two were reports published by professional organizations [25, 26]. Despite examining the same topic, each study used different search criteria to find pain treatment facilities and used different eligibility criteria for what was considered a pain treatment facility (see Table 1). The response rate in studies ranged from 56% to 100% (M = 80.1%; 95% CI: 70.92 to 89.31). Each study has a bias according to the time it was conducted, location of the survey, the manner in which it was published (i.e., in a peer-reviewed journal or an independently published report or project), pain treatment facility search strategy, eligibility criteria, and response rate.

All studies used a survey that was sent by phone, fax, email, or mail. Only one study [20] included an interview as a part of data collection. Pain treatment facility directors, chief executive officers, head anesthesiologists, and, in one case, senior psychologists were asked to complete the questionnaire. Surveys were developed for each study with two exceptions: Peng et al. [17, 18, 21] used an adapted version of the Québec Chronic Pain Clinic Survey developed by Veillette et al. [22] and Csordas and Clark [30] did not specify what survey was used.

Health care systems can vary substantially depending on the country of origin [32]. For this reason, the results on pain facility characteristics are reported by country (Australia, Canada, Italy, United Kingdom, and United States).

### 3.3. Availability and Caseloads of Pain Treatment Facilities

Data on the availability, caseloads, and wait times for pain treatment facilities are listed in Tables 2 and 3. In many cases, the metric used to report this information varied, with some studies reporting medians, means, ranges, or sums. To facilitate meaningful comparisons across studies, relevant data were transformed to match the metrics reported in other studies (e.g., if new consultation appointments were reported annually, it was divided by 12 to permit comparison to other articles that reported this index according to new consultations per month).

*Australia*. In their survey, Hogg et al. [20] found that there was only one pain treatment facility for 310,000 Australians. The authors reported that new consultation appointments ranged from 24.92 to 36.75 per month. The median wait time for public facilities was reported to be 150 days, with the median wait time of private facilities being 38.5 days. The reported median for all facilities was 103 days.

*Canada*. Only Peng et al. [18] reported on the proportion of pain facilities for the population, finding that there is one pain treatment facility for 258,000 people in Canada. They found that the average number of monthly consultations and wait times varied according to the type of facility surveyed. In pediatric pain treatment facilities [21], fewer new consultations (Mdn = 2.58) and follow-up appointments (Mdn = 37.50) were reported and the median wait time for all facilities was 28 days, with a median of 10 patients waiting for treatment. More appointments (new: M = 37.58, follow-up: M = 432.19) were reported for adult pain treatment facilities [18], with the median wait time reported being 180 days for public facilities and 15 days for private facilities. In hospital anesthesia departments in Québec [22], the reported caseload was M = 16 for new consultations and M = 74 for follow-up appointments, with 4500 patients awaiting treatment.

*Italy*. Only one measure of caseload was available for Italy. De Benedittis and Lorenzetti [23] reported M = 164.58 appointments per month for the 63 pain treatment facilities surveyed. No information was available regarding access to care or wait times.

*United Kingdom*. The National Pain Audit [27] estimated that there was one pain treatment facility for 200,000–370,370 people living in the United Kingdom. The number of appointments varied [new: average = 35, follow-up: average = 21 [25]]. The Clinical Standards Advisory Group [25] reported wait times according to the type of care needed. The longest median wait time was reported for routine care (112 days), while the median wait time for urgent care was 14 days and only 7 days for cancer care. The report by Dr. Foster & the Pain Society [26] indicated that the median wait times were longer for patients referred by consultants (161 days) in comparison to general practitioners (140 days). The National Pain Audit [27] reported that wait times for 80% of English facilities were less than 126 days, although only 50% of Welsh pain facilities had wait times meeting these criteria.

*United States*. Information on caseload was available for two studies. Castel et al. [29] reported that pain treatment facilities in North Carolina had a high number (M = 1244.10) of appointments per month. In 1985, Hickling et al. [31] reported new consultations (M = 36.1) and follow-up appointments (M = 74.3) to be significantly lower. No information was available regarding access to care or wait times.

### 3.4. Treatments Available at Pain Services

A wide range of medical, physical, psychological, and other treatments were reported at pain treatment facilities (see Tables 4 [Table tab5]–6).

*Australia*. Only one form of pain treatment was reported by Hogg et al. [20]. They reported that 3.5% of pain treatment facilities used acupuncture.

*Canada*. In three Canadian surveys [18, 21, 22], medical treatments included cryotherapy (6%), injections [trigger point injection (60–88%), botulinic toxin injection (26–44%), continuous epidural (80%), one-shot epidural or epidural injection (46–100%), facet joint/nerve injection (51–60%), and intravenous regional anaesthesia (31–82%)], nerve blocks [caudal block (37–74%), paravertebral nerve block (40–48%), peripheral nerve block (49–90%), radiofrequency lesioning (40%), stellate ganglion nerve block (42–92%), and sympathetic block/local anesthetic (32–52%)], pharmacotherapy (100%), and spinal cord stimulation (12%). Physical therapy related treatments included acupuncture (40–53%), exercise programs (77–100%), hydrotherapy (35–100%), intramuscular stimulation (20–30%), massage (20–34%), physiotherapy (39–100%), and transcutaneous electrical nerve stimulation (66–80%). Psychological treatments included biofeedback (39–80%), couples therapy (25%), family therapy (25–60%), group psychotherapy (20–42%), hypnosis (28–80%), imagery (50–100%), individual psychotherapy (79–100%), relaxation/breathing (71–100%), and support groups (40–42%). Other treatments included dietary/nutrition counseling (40–47%) and pharmaceutical counseling (37–40%).

*Italy*. De Benedittis and Lorenzetti [23] reported a mean utilization index instead of the percentage of facilities that used specific treatments and data was therefore not included in the tables. Of treatments that were reported in at least one other study, they reported that pain treatment facilities used nerve blocks, percutaneous discectomies, pharmacotherapy (including psychotropic medication, long-acting narcotics/opioids, and other medications), and spinal cord stimulation.

*United Kingdom*. Three surveys in the United Kingdom reported on treatments used in pain treatment facilities [24–26]. Medical treatments included cryotherapy (49–68.8%), injections [73%: continuous epidural (61–73%) and one-shot epidural or epidural injections (95-96%)], nerve blocks [99%: radiofrequency lesioning (51–65%) and sympathetic block/local anesthetic (81.3%)], long-acting narcotics/opioid medications (75–91%), and spinal cord stimulation (26–38%). Physical therapy related treatments included acupuncture (87.5–90%), physiotherapy (57–82%), and transcutaneous electrical nerve stimulation (94–100%). Psychological treatments included hypnosis (6.3–20%) and individual psychotherapy (66–69%). Other treatments included homeopathy (4.5%).

*United States*. Medical treatments included injections [trigger point injections (4–87%), one-shot epidural or epidural injections (76%), and facet joint/nerve injections (76%)], nerve blocks [caudal block (36–70.7%)], pharmacotherapy [89%: antidepressants/psychotropics (40–82.7%), analgesics (24–74%), and long-acting narcotics/opioids (74%)], surgery (20–34.7%), and percutaneous discectomies (20%). Physical therapy related treatments included acupuncture (8–22%), exercise programs (4–48%), massage (15-16%), physiotherapy (56%), and transcutaneous electrical nerve stimulation (20–86.7%). Psychological treatments included biofeedback (48–85.3%), couples therapy (72%), education (22–24%), family therapy (68%), hypnosis (20%), individual psychotherapy (22–92%), and relaxation/breathing (48%). Other treatments included dietary/nutrition counseling (12–35%) and homeopathy (20%).

### 3.5. Attributes of Pain Treatment Facilities

Data on the attributes of pain treatment facilities, including staff composition, space and equipment available, administrative support, and multidisciplinary status, are presented in Tables 7 and 8.

*Australia*. Information on pain professionals working at pain treatment facilities was not reported by Hogg et al. [20], although information on space and multidisciplinary status was available. It was reported that 60% of pain treatment facilities had inpatient care/beds [20]. The authors reported that 46% of pain facilities met Level 1 IASP criteria [14] (i.e., a multidisciplinary pain center) and 33% met Level 2 criteria (i.e., a multidisciplinary pain clinic). Additionally, 33.3% of facilities offered training and 74% offered a pain management program (PMP) [20].

*Canada*. Three Canadian studies reported on the most frequently reported pain professionals working at pain treatment facilities [17, 21, 22]: anesthesiologists (51–100%), nurses (57–100%), physiotherapists (10–80%), and psychologists (13–100%). Two studies [17, 21] reported on psychiatrists (20–22%). Other reported pain professionals included acupuncturists, dentists, general practitioners, neurologists, neurosurgeons, occupational therapists, orthopedic surgeons, physiatrists, respiratory care therapists, rheumatologists, and social workers. Data on space, equipment, and staff at pain treatment facilities was only reported by Veillette et al. [22]. They reported that 52% had consultation/treatment room(s), 93.8% had an operating theatre, 48% had recovery rooms, 75% had hospital/outpatient beds, and 77.6% had fluoroscopy equipment. Peng et al. [21] reported that 76% of adult pain treatment facilities offered training and 64% conducted research, with 39% meeting IASP Level 2 criteria for multidisciplinary status (i.e., as multidisciplinary pain clinics, as reported by Peng et al. [17]). Peng et al. [18] reported that 100% of pediatric pain treatment facilities offered training and 60% conducted research.

*Italy*. De Benedittis and Lorenzetti [23] reported that the following health care professions were employed: anesthesiologists (71.4%), internal medicine specialists (23.8%), neurologists (36.5%), neurosurgeons (20.6%), occupational therapists (3.2%), physiatrists (14.3%), psychiatrists (22.2%), psychologists (30.2%), and social workers (12.7%). The majority of pain treatment facilities reported having an outpatient clinic (80.9%), a nerve block room (57.1%), and an operating theatre (50.8%). Inpatient care was reported in 41.3% and a day hospital in 44.4%. Multidisciplinary status of pain treatment facilities was not assessed, although it was reported that 76.2% of facilities offered training and 79.4% conducted research.

*United Kingdom*. Three studies reported on the staffing of pain professionals [24, 25, 27]. The most frequently reported were pharmacists (7–100%), physiotherapists (52–100%), psychologists (18.8–67%), consultants (71–91%), and nurses (66.4–93.8%). Data on space, equipment, and staff were variable. The most commonly reported attribute was access to inpatient care (24–93.8%). Other space, access, and equipment data reported included the availability of consultation rooms (79%), operating theatres (81%), hospital/outpatient beds (87.5%), office space for administrative staff (80.3–83%), office space for professional staff (66–84%), wheelchair access (80–100%), and X-ray imaging (89–93.8%). The National Pain Audit [27] was the only survey published in the United Kingdom that reported data on the multidisciplinary status of pain treatment facilities, finding that 40% of English and 60% of Welsh facilities met IASP Level 2 criteria (i.e., as multidisciplinary pain clinics). Pain management programs were offered by 40% [25] and 58% [26]. The Clinical Standards Advisory Group [25] reported that 36% of facilities conducted research and 100% of multidisciplinary pain centers surveyed in the National Pain Audit [28] conducted research. National Pain Audit [27] reported that clinical training was provided to medical students, physiotherapy students, and nurses (range: 53–80%) and that research was conducted at 27% of English facilities and 20% of Welsh facilities.

*United States*. Three surveys [29–31] reported on the incidence of employed physiotherapists (26–75%), psychiatrists (26–80%), and psychologists (22–85.5%). Two surveys [29–31] reported on the incidence of acupuncturists (2.6–50%), anesthesiologists (57–59.2%), general practitioners (1.3–7%), internal medicine specialists (0–31.6%), neurologists (9–46.1%), neurosurgeons (16–56.6%), nurses (30–63.2%), occupational therapists (47.4–56%), orthopedic surgeons (18–40.8%), pharmacists (2–2.6%), and physiatrists (33–42.1%). One survey reported on the employment of dentists [22.4% [31]], respiratory care therapists [1.3% [31]], rheumatologists [2% [29]], and social workers [47.4% [31]]. No American surveys reported data on space, equipment, or support staff. One study by Castel et al. [29] reported on the multidisciplinary status of pain treatment facilities, finding that only 4.3% met IASP Level 2 criteria (i.e., as multidisciplinary pain clinics), although Hickling et al. [31] reported that 76.3% of surveyed facilities offered clinical training. Other studies did not use an existing classification for multidisciplinary status. For example, Csordas and Clark [30] reported that 28% of pain facilities were comprehensive, 20% were syndrome specific, 8% were modality specific, 36% were partial clinics, and 8% were combined with other departments.

## 4. Discussion

The present study systematically reviewed the literature on surveys of pain treatment facilities. Our search yielded 14 English language studies published between 1985 and 2013. Surveys were conducted in Australia, Canada, Italy, the United Kingdom, and the United States. The results reveal little consistency in the research design and questionnaires used to describe pain treatment facilities. Each study used different search and inclusion criteria for pain treatment facilities. The availability of pain treatment facilities was scarce even though the caseloads and wait times for facilities were generally high. A wide range of interventional medical, physical, and psychological pain treatments were available. Most studies reported findings on the percentage of practitioners in different health care professions employed. Taken together, these findings suggest that there is considerable variability in the availability, services, and professionals of multidisciplinary pain clinics.

Each survey used a different approach to identify pain treatment facilities (Table 1). In some cases, facilities were identified through contacting individuals with membership in a specific association (e.g., [24]), searching the Yellow Pages (e.g., [29]), consulting a published list of facilities (e.g., [25, 31]), or using a combination of different strategies. When the search for pain treatment facilities is not comprehensive, there is a risk of underestimating the availability of pain treatment facilities in a region and of selection bias that may undermine the representativeness of sampled pain facility characteristics. For example, Veillette et al. [22] identified pain treatment facilities by contacting the heads of anesthesia departments, a strategy that may have omitted facilities that are not directed by individuals in that role. This suggests that caution must be taken when interpreting and generalizing findings.

Pain treatment facilities are both scarce and in high demand (Tables 2 and 3). Access to multidisciplinary care varied by country, with one per 310,000 people in Australia [20], one per 258,000 people in Canada [18], and one per 200,000–370,370 people in the United Kingdom [27]. With an estimated 37% of the world population suffering from chronic pain [1], this reflects poor availability of services for pain sufferers. The demand for multidisciplinary pain treatment facilities is reflected in the wait times.

In 2005, IASP [33] developed recommendations for wait times based on an international task force of experts. They suggest wait times for acute painful conditions to be immediate, most urgent pain to be within one week, urgent or semiurgent pain to be within one month, and routine or regular pain to be within eight weeks. In the present study, we found one article from the United Kingdom that reported on wait times according to the severity of painful conditions [25] that showed the median wait times to be two weeks for urgent care and 16 weeks for routine care, both substantially longer than the suggested wait times. Two surveys showed breakdown of wait times according to public and private facilities [18, 20]. It was found that wait times were longer in public facilities in Australia (Mdn = 21.4 weeks) and Canada (Mdn = 25.7) in comparison to private facilities (Australia: Mdn = 5.5, Canada: Mdn = 2.1), showing that those that can afford to pay out of pocket for medical services can receive multidisciplinary pain treatment faster. No information was available on wait times from surveys conducted in the United States, where public funding is mainly limited to individuals that are 65 years old and older, individuals with disabilities, and some individuals with low income, although there are substantial gaps in health care coverage for the general American population [34]. It is possible that, in the United States, there may be increased demand for private services that may increase wait times. Importantly, it appears that access to the multidisciplinary care may be faster in pediatric settings: in a survey of five pediatric pain treatment facilities, Peng et al. [21] reported the median wait time to be four weeks, although it was not specified whether these facilities were public or private. Taken together, as longer wait times are associated with deterioration of health and lowered quality of life [35], the present findings suggest an alarming global situation in which access to pain treatment facilities requires improvement.

Unsurprisingly, it was found that medical pain treatments were reported more frequently in pain treatment facilities in acute care hospitals in Québec [22] in comparison to pain treatment facilities not restricted to hospital settings that were surveyed across Canada [18, 21]. The emphasis on medical treatments by Veillette et al. [22] is also illustrated in the exclusion of data collected on the prevalence of physical therapy and psychological and other treatments that were reported in Canada-wide surveys [18, 21]. Surveys conducted in the United Kingdom were published between 1988 and 2003. Despite the range, the prevalence of some reported treatments did not vary greatly. For example, continuous epidural was reportedly used in 68.8% of facilities in Bisset [24], 73% in Clinical Standards Advisory Group [25], and 61% in the report by Dr. Foster & the Pain Society [26]. However, it is noteworthy that only treatments reported in at least two studies (regardless of country of origin) were included in the review. In the three surveys conducted in the United States, the prevalence of injections and nerve blocks was only reported in two studies, with more information reported on pharmacotherapy treatments. Little information was available on pain treatments for surveys conducted in Australia and Italy, limiting the discussion on their use of interventions in pain treatment facilities.

According to IASP, multidisciplinary pain centers and clinics must be staffed by a variety of health care professionals that include physicians, nurses, mental health professionals, and physical therapists [14]. All Canadian surveys reported on the prevalence of these employed professionals, even though they were not always represented. However, in pediatric settings, Peng et al. [21] found that 100% of pain treatment facilities staffed a psychologist and a nurse, while 80% staffed an anaesthesiologist and physiotherapist. In Italy, the majority of pain facilities surveyed by De Benedittis and Lorenzetti [23] did not satisfy the staff requirements of a multidisciplinary pain centre or clinic given that physical therapists were reported in only 14.3% of facilities and mental health professionals were reported in 30.2%. Data on the staff composition from surveys in the United Kingdom did not include any information on employed physicians, although the authors reported that nurses were staffed in up to 93.8% of facilities [24], physiotherapists in up to 100% [24], and psychologists in up to 67% [25]. In surveys conducted in the United States, Castel et al. [29] found that only a minority of facilities met IASP criteria for a multidisciplinary clinic and this low number was attributed to only having one provider type on staff. This may be related to the methodology used in this survey, as only pain facilities that specialized in treating neck and back pain were contacted for the survey. Although the staff composition was not surveyed by Hogg et al. [20], they reported that 46% of pain treatment facilities in Australia met IASP criteria for a multidisciplinary pain centre. Overall, even though many studies did not include a measure of the multidisciplinary functionality, an assessment of facilities provided demonstrates that pain treatment facilities attempt to provide comprehensive treatment to target both biomedical and psychological dimensions of pain.

This study has several limitations. The data extracted from the studies included in the review were heterogeneous, with surveys completed over a 28-year period and in five countries. There are considerable differences in what is considered to be a pain treatment facility and the importance of various treatment strategies has changed over time [14, 36]. The method of identifying pain treatment facilities and the differing eligibility criteria introduced bias where not all multidisciplinary pain clinics may have been included in the reported surveys. This may have been similarly affected by the responses rate, which ranged between 56 and 100%. Additionally, drawing inferences across the extracted data sample was challenging due to the extent of missing data and differently reported metrics.

Regular, comprehensive surveys and audits of pain treatment facilities are warranted. They provide important information on the availability of services, wait times to access treatment, and the types of services available. There were also many variables of interest for future research that were assessed in some studies but not others (see Table 9). These include information regarding the cost of treatments, outcome measures used, whether facilities are public or private, and guidelines that are currently in use. Future investigations should consider these variables. The Canadian Pain Coalition conducted this study as a part of the first phase of the Report Card on Pain. For the second phase, the Report Card on Pain aims to complete an updated survey of pain treatment facilities in Canada, as the most recent survey completed was over 8 years ago [18].

In summary, this systematic review of studies surveying pain treatment facilities around the world highlights important information for policymakers on the scope, demand, and accessibility of such facilities and provides direction for future researchers to enhance the comprehensiveness and comparability of data capture. The results showed that there is a great deal of diversity in the information collected and future studies should consider using more comprehensive search strategies, increasing clarity on what is considered to be a multidisciplinary pain treatment facility, and reporting on a consistent set of variables to provide a clear summary of the status of multidisciplinary pain treatment facilities. Despite their variability, the identified studies shed light on substantial gaps in tackling the challenge of pain and underscore the pressing need for improved access to pain treatment facilities globally.

## Summary

This study systematically reviewed the published literature describing multidisciplinary chronic pain treatment facilities to provide an overview of their availability and characteristics. Fourteen articles satisfied inclusion criteria (i.e., original research that directly surveyed a range of treatments in more than one pain treatment facility). Results show considerable variability in the research methodologies used to describe pain treatment facilities, show that availability of pain treatment facilities was scarce, and show that accompanying caseloads and wait times were generally high. Pain treatment facilities used a wide range of pain interventions and employed a variety of health care professionals. Although the present study was limited by the heterogeneous data extracted from included studies, it highlights important information on the scope, demand, and accessibility of multidisciplinary pain treatment facilities.

## Figures and Tables

**Table 1:** List and details of papers included in the systematic review.

Country of study (specific region)	Author(s) (year)	Number of facilities that responded (% of total)	Search strategy to identify pain treatment facilities	Eligibility criteria of pain treatment facilities sampled
Australia (countrywide)	Hogg et al. (2012) [20]	57 (86%)	(1) A list of pain management services available from the Australian Pain Society was consulted (2) Internet searches (3) Australian region leaders were consulted to identify pain services	(1) Must serve at least 100 clients per annum (2) Pain centers offering focused treatment on children and adults were included

Canada (countrywide)	Peng et al. (2007) [18]	102 (85%)	(1) Letters sent to medical directors/chief executive officers at all Canadian hospitals and rehabilitation centers (2) Provincial compensation agencies, insurance bureau of Canada, and pharmaceutical industry representatives contacted (3) List reviewed by provincial representatives involved in the study and insured eligibility as a MPTF	(1) Advertise as a pain clinic or pain center or offer multidisciplinary pain services (2) Staff must include at least three different types of health care disciplines, one of which must be medical

Canada (countrywide)	Peng et al. (2007) [21]	5 (100%)	The same search protocol as Peng et al. (2007) [18]	(1) Advertise as a pain clinic, pain center, or multidisciplinary pain services (2) Staff must include at least three different types of health care disciplines, one of which must be medical (3) Pain services must exclusively be offered to children up until the age of 18

Canada (countrywide)	Peng et al. (2008) [17]	102 (85%)	The same search protocol as Peng et al. (2007) [18]	The same eligibility criteria as Peng et al. (2007) [18]

Canada (Québec)	Veillette et al. (2005) [22]	50 (100%)	(1) Heads of anaesthesia departments were contacted at all acute care hospitals in Québec (2) Pain centers serving pediatric populations or without permanent anesthesiologists were excluded	(1) The anaesthesia departments contacted must offer treatment for patients with chronic noncancer pain

Italy (countrywide)	De Benedittis and Lorenzetti (1989) [23]	63 (58%)	Search strategy not reported	Eligibility criteria not reported

United Kingdom (Scotland)	Bisset (1988) [24]	16 (unclear)	(1) All known consultant anaesthetic members of the North British Pain Association in Scotland were contacted (2) If no anaesthetic member was listed for a hospital, a known consultant was contacted (3) If no anesthetic member or known consultant was listed/available, a listed anaesthetist in the register at that hospital was contacted	(1) Pain relief clinics under anaesthetic management

United Kingdom (countrywide)	Clinical Standards Advisory Group (2000) [25]	121 (56%)	(1) All National Health Service Trusts in the United Kingdom as listed in Binley's Directory of NHS management (1997) were contacted	(1) All sites were required to be a National Health Service Trust (2) Sites had to provide one or more pain services

United Kingdom^1^ (countrywide)	Dr. Foster & the Pain Society (2003) [26]	161 (76%)	(1) Distributed to a list of member contacts provided by the Pain Society at 214 hospitals across the United Kingdom	(1) Hospital must have a pain service

United Kingdom (England and Wales)	National Pain Audit (2011) [27]	214 (unclear)	(1) Contacts emailed in the following settings: within Primary Care Trusts, Local Health Boards, Hospital Audit Leads, PCT audit leads, and British Pain Society members (2) Letters sent to all chief executives of the Chief Medical Officer (3) Advertisements placed in the chief executives bulletin, the British Pain Society Newsletter, and Faculty of Pain Medicine section of the Royal College of Anaesthetists Bulletin (4) Hospital services using treatment definition code 191	(1) Any service that met the Hospital Episode Statistics treatment definition of a specialist pain management site (2) No restrictions were made according to clinic settings

United Kingdom (England and Wales)	National Pain Audit (2013) [28]	121 (66%)	(1) List of clinics obtained from the National Health Service Choices website	Eligibility criteria not reported

United States (North Carolina)	Castel et al. (2009) [29]	46 (74%)	(1) North Carolina residents with chronic back and/or neck pain were surveyed (2) Searching North Carolina Yellow Pages listings electronically	(1) Clinics had to treat patients experiencing chronic pain lasting at least three months (2) Patients must be suffering from back and neck pain (3) Pain services with only one specialty were not sampled in the Yellow Pages electronic search

United States (region not specified)	Csordas and Clark (1992) [30]	25 (93%)	(1) Reviewing advertisements and news features, centers inviting research (2) Searching lists published in magazines and directories (3) Inquiries/referrals from medical professionals and colleagues (4) Asking survey responders about other clinics	(1) Must identify itself as a pain center or offer a pain program

United States (countrywide)	Hickling et al. (1985) [31]	76 (66%)	(1) Consulting the pain directory of the American Society of Anesthesiologists published in 1979	Eligible clinics had to meet 8 out of 11 criteria developed by the American Society of Anesthesiologists directory (1979): (1) Space and beds exclusively available to the pain service (2) Staffed by full-time professional staff of at least two disciplines (3) Full-time supportive staff (4) Process for screening and selection of patients (5) Review and maintenance must be recorded (6) Consultants from multiple disciplines (7) Routine psychological assessment (8) Research activities that are ongoing (9) Training program (10) Therapy able to target the physical and psychosocial problems of patients (11) Periodic outcome evaluation

^1^Funded by an educational grant from Napp Pharmaceuticals Ltd.

**Table 2:** Availability and caseload of pain treatment facilities.

Author(s) (year)	Pain facility per population	New consultation appointments per facility per month	Follow-up appointments per facility per month	Appointments per facility per month
Australia				
Hogg et al. (2012) [20]	1 per 310,000 people	Range = 24.92–36.75^a^	—	—
Canada				
Peng et al. (2007) [21]	—	Mdn = 2.58^a^	Mdn = 37.50^a^	—
Peng et al. (2007) [18]	1 per 258,000 people	M = 37.58^ab^	M = 432.19^ab^	—
Veillette et al. (2005) [22]	—	M = 16^b^	M = 74^b^	—
Italy				
De Benedittis and Lorenzetti (1989) [23]	—	—	—	M = 164.58^a^
United Kingdom				
Clinical Standards Advisory Group (2000) [25]	—	Average = 35	Average = 21	—
Dr. Foster & the Pain Society (2003) [26]	—	—	—	Range = 15–750^a^
National Pain Audit (2011) [27]	Range = 0.27–0.50 per 100,000 people	—	—	—
United States				
Castel et al. (2009) [29]	—	—	—	M = 1244.10^c^, Mdn = 709.05^c^
Hickling et al. (1985) [31]	—	M = 36.1	M = 74.3	—

*Note*. Information on incidence and caseload was not available for Bisset [24], Csordas and Clark [30], National Pain Audit [28], and Peng et al. [17].

^a^Divided by 12 to provide a monthly estimate since annual appointments were reported in source paper.

^b^Divided by the number of pain treatment services surveyed since the total number of appointments was reported in the source paper.

^c^Multiplied by 4.35 to provide a monthly estimate since weekly appointments were reported in source paper.

**Table 3:** Pain treatment facility wait times and number of patients waiting for treatment.

Author(s) (year)	Public facilities, median (interquartile range)	Private facilities, median (interquartile range)	All facilities, median (interquartile range)	Number of patients, median (interquartile range)
Australia				
Hogg et al. (2012) [20]	150 days (68–281)^a^	38.5 days (24–75)^a^	103 days (44–210)^a^	—
Canada				
Peng et al. (2007) [18]	180 days (60–420)^b^	15 days (9–30)^b^	—	—
Peng et al. (2007) [21]	—	—	28 days (14–42)^a,b^	10 patients (2–17)
Veillette et al. (2005) [22]	—	—	—	4500 patients, total^c^
United Kingdom	—	—		
Clinical Standards Advisory Group (2000) [25]	—	—	—	90 patients (45–150)^d^
Routine care	—	—	112 days (70–196)^b,e^	—
Urgent care	—	—	14 days (7–14)^b,e^	—
Cancer care	—	—	7 days (7–14 days)^b,e^	—
Dr. Foster & the Pain Society (2003) [26]	—	—	—	—
Referred by general practitioners	—	—	140 days (total range: 28–770)^b^	—
Referred by consultants	—	—	161 days (total range: 28–931)^a^	—
National Pain Audit (2011) [27]	—	—	—	—
England	—	—	80% of services under 126 days^b^. If above, Mdn = 140 days^b^	—
Wales	—	—	50% of services under 126 days^b^. If above, Mdn = 231 days^b^	—

*Note*. Information on wait times and patients waiting for treatment was not available for Bisset [24], Castel et al. [29], Csordas and Clark [30], De Benedittis and Lorenzetti [23], Hickling et al. [31], National Pain Audit [28], and Peng et al. [17].

^a^Initial/first assessments.

^b^Converted from weeks or months to days.

^c^Of this group, 67% were waiting for longer than 9 months.

^d^New and old patients.

^e^New outpatients.

**Table 4:** Medical treatments used at pain treatment facilities.

Treatment	Canada				United Kingdom				United States		
	Peng et al. (2007) [18]	Peng et al. (2007) [21]	Veillette et al. (2005) [22]		Bisset (1988) [24]	Clinical Standards Advisory Group (2000) [25]	Dr. Foster & the Pain Society (2003) [26]		Castel et al. (2009) [29]	Csordas and Clark (1992) [30]	Hickling et al. (1985) [31]
Cryotherapy	—	—	6%		68.8%	—	49%		—	—	—
Injections	—	—	—		—	—	73%		—	—	—
Trigger point injection	63%	60%	88%		—	—	—		87%	4%	—
Botulinic toxin injection	44%	40%	26%		—	—	—		—	—	—
Continuous epidural	—	80%	—		68.8%	73%	61%		—	—	—
One-shot epidural or epidural injection	46%	60%	100%		—	96%	95%		76%	—	—
Facet joint/nerve injection	51%	60%	—		—	—	—		76%	—	—
Intravenous regional anaesthesia	31%	40%	82%		—	—	—		—	—	—
Nerve blocks	—	—	—		—	99%	—		—	36%	70.7%
Caudal block	37%	—	74%		—	—	—		—	—	—
Paravertebral nerve block	44%	40%	48%		—	—	—		—	—	—
Peripheral nerve block	49%	60%	90%		—	—	—		—	—	—
Radiofrequency lesioning	—	40%	—		—	51%	65%		—	—	—
Stellate ganglion nerve block	42%	60%	92%		—	—	—		—	—	—
Sympathetic block/local anesthetic	32%	40%	52%		81.3%	—	—		—	—	—
Pharmacotherapy	—	—	100%		100%	—	—		89%	—	—
Antidepressants/psychotropics	—	—	—		—	—	—		67%	40%	82.7%
Analgesics	—	—	—		—	—	—		72%	24%	—
Long-acting narcotics/opioids	—	—	—		—	75%	91%		74%		—
Spinal cord stimulation	—	—	12%		—	26%	38%		50%	—	—
Surgery	—	—	—		—	—	—		—	20%	34.7%
Percutaneous discectomy	—	—	—		—	—	—		20%	—	—

*Note*. Information on medical treatments was not available for National Pain Audit [27], [28] and Peng et al. [17]. Hogg et al. [20] did not state specific procedures but indicated that 87.7% of pain treatment facilities used some sort of interventional medicine procedure. De Benedittis and Lorenzetti [23] reported a mean utilization index instead of the percentage of facilities that used a specific procedure and data were therefore not included in the table. Articles reporting on a procedure not reported in another study were not included in the table (*n* = 27 procedures). Articles reporting treatments without percentages were not included.

**Table 5 tab5:** Physical therapy related treatments used at pain treatment facilities.

Treatment	Australia	Canada			United Kingdom				United States		
	Hogg et al. (2012) [20]	Peng et al. (2007) [18]	Peng et al. (2007) [21]		Bisset (1988) [24]	Clinical Standards Advisory Group (2000) [25]	Dr. Foster & the Pain Society (2003) [26]		Castel et al. (2009) [29]	Csordas and Clark (1992) [30]	Hickling et al. (1985) [31]
Acupuncture	3.5%	53%	40%		87.5%	86%	90%		22%	8%	—
Exercise program	—	77%	100%		—	—	—		48%	4%	—
Hydrotherapy	—	35%	100%		—	—	—		—	—	—
Intramuscular stimulation	—	30%	20%		—	—	—		—	—	—
Massage	—	34%	20%		—	—	—		15%	16%	—
Physical/physiotherapy	—	—	—		—	82%	—		—	56%	—
Individual	—	75%	100%		—	—	79%		—	—	—
Group	—	39%	—		—	—	57%		—	—	—
Transcutaneous electrical nerve stimulation	—	66%	80%		100%	98%	94%		52%	20%	86.7%

*Note*. Information on physical therapy related treatments was not available for National Pain Audit [27], [28], Peng et al. [17], and Veillette et al. [22]. De Benedittis and Lorenzetti [23] reported a mean utilization index instead of the percentage of pain treatment facilities that used a specific procedure and data were therefore not included in the table. Articles reporting on a procedure not reported in another study were not included in the table (*n* = 11 procedures). Articles reporting treatments without percentages were not included.

**Table 6:** Psychological treatments and other treatments used at pain treatment facilities.

Treatment	Canada			United Kingdom				United States		
	Peng et al. (2007) [18]	Peng et al. (2007) [21]		Bisset (1988) [24]	Clinical Standards Advisory Group (2000) [25]	Dr. Foster & the Pain Society (2003) [26]		Castel et al. (2009) [29]	Csordas and Clark (1992) [30]	Hickling et al. (1985) [31]
Psychological treatments										
Biofeedback	39%	80%		—	—	—		—	48%	85.3%
Couple therapy	25%^a^	—		—	—	—		—	—	72%
Education	—	—		—	—	—		22%	24%	—
Family therapy	25%^a^	60%		—	—	—		—	—	68%
Group psychotherapy	42%	20%		—	—	—		—	—	—
Hypnosis	28%	80%		6.3%	20%	—		—	20%	—
Imagery	50%	100%		—	—	—		—	—	—
Individual psychotherapy	79%	100%		—	66%	69%		22%	80%	92%
Relaxation/breathing	71%	100%		—	—	—		—	48%	—
Support group	42%	40%		—	—	—		—	—	—
Other treatments										
Dietary/nutrition counseling	47%	40%		—	—	—		35%	12%	—
Homeopathy	—	—		—	4.5%	—		20%	—	—
Pharmaceutical counseling	37%	40%		—	—	—		—	—	—

*Note*. Information on psychological and other treatments was not available for Hogg et al. [20], National Pain Audit [27], [28], Peng et al. [17], and Veillette et al. [22]. De Benedittis and Lorenzetti [23] reported a mean utilization index instead of the percentage of pain treatment facilities that used a specific procedure and data were therefore not included in the table. Articles reporting on a procedure not reported in another study were not included in the table (*n* = 17 procedures). Articles reporting treatments without percentages were not included.

^a^Reported as family/couple therapy.

**Table 7:** Pain professionals working at pain treatment facilities.

Treatment	Canada				Italy		United Kingdom				United States		
	Peng et al. (2008) [17]	Peng et al. (2007) [21]	Veillette et al. (2005) [22]		De Benedittis and Lorenzetti (1989) [23]		Bisset (1988) [24]	Clinical Standards Advisory Group (2000) [25]	National Pain Audit (2011), England/Wales [27]		Castel et al. (2009) [29]	Csordas and Clark (1992) [30]	Hickling et al. (1985) [31]
Acupuncturist	35%	—	—		—		—	—	—		20%	—	2.6%
Anesthesiologist	51%	80%	100%		71.4%		—	—	—		57%	—	59.2%
Consultant	—	—	—		—		—	91%	71%/90%^a^		—	—	—
Dentist	6.9%	—	—		—		—	—	—		—	—	22.4%
General practitioner	56%	—	—		—		—	—	—		7%	—	1.3%
Internal medicine specialist	—	—	—		23.8%		—	—	—		0%	—	31.6%
Neurologist	13%	—	—		36.5%		—	—	—		9%	—	46.1%
Neurosurgeon	7%	—	—		20.6%		—	—	—		16%	—	56.6%
Nurse	57%	100%	71%		—		93.8%	66.4%	—		30%	—	63.2%
Occupational therapist	—	—	0%		3.2%		—	57%	—		—	56%^b^	47.4%
Orthopedic surgeon	14%	—	—		—		—	—	—		18%	—	40.8%
Pharmacist	—	—	—		—		100%	7%	78%/30%		2%	—	2.6%
Physiatrist	32%	—	—		14.3%		—	—	—		33%	—	42.1%
Physiotherapists	75%	80%	10%		—		100%	80%	52%/60%		26%	56%^b^	75%
Psychiatrist	22%	20%	—		22.2%		—	—	—		26%	80%^c^	59.2%
Psychologist	68%	100%	13%		30.2%		18.8%	67%	48%/60%		22%	80%^c^	85.5%
Respiratory care therapist	—	—	42%		—		—	—	—		—	—	1.3%
Rheumatologist	9%	—	—		—		—	—	—		2%	—	—
Social worker	—	—	0%		12.7%		—	—	—		—	—	47.4%

*Note*. Information on employed pain professionals was not available for Dr. Foster & the Pain Society [26], Hogg et al. [20], National Pain Audit [28], and Peng et al. [18]. Articles reporting on a profession not reported in another study were not included in the table (*n* = 29 professions). Articles reporting professions without a percentage were not included.

^a^Reported only for services with specialist medication management.

^b^Listed in the paper as either a physical or occupational therapist.

^c^Listed in the paper as either psychiatrists or psychologists.

**Table 8:** Multidisciplinary status of pain treatment facilities.

Author(s) (year)	% of facilities offering training	% of facilities conducting research	% of facilities meeting IASP criteria by level		% of facilities offering a PMP
			1	2	
Australia					
Hogg et al. (2012) [20]	33.3%	—	46%^*∗*^	33%^*∗*^	74%
Canada					
Peng et al. (2007) [18]	76%	64%	—	—	—
Peng et al. (2007) [21]	100%	60%	—	—	—
Peng et al. (2008) [17]	—	—	—	39%^*∗∗*^	—
Italy					
De Benedittis and Lorenzetti (1989) [23]	76.2%	79.4%	—	—	—
United Kingdom					
Clinical Standards Advisory Group (2000) [25]	—	36%	—	—	40%
Dr. Foster & the Pain Society (2003) [26]	—	—	—	—	58%
National Pain Audit (2011) [27]	—	—	—	—	—
England	53%^a^/66%^b^	27%	—	40%^*∗∗∗*^	—
Wales	50%^c^/70%^d^/80%^b^	20%	—	60%^*∗∗∗*^	—
National Pain Audit (2013) [28]	—	100%^e^	—	—	—
United States					
Castel et al. (2009) [29]	—	—	—	4.3%^*∗∗*^	—
Hickling et al. (1985) [31]	76.3%	—	—	—	—

*Note*. Information on multidisciplinary status was not available for Bisset [24], Csordas and Clark [30], and Veillette et al. [22].

IASP: International Association for the Study of Pain.

PMP: pain management program.

^a^Training for medical and physiotherapy students.

^b^Training for nurses.

^c^Training for medical students.

^d^Training for physiotherapist students.

^e^Reported only for facilities considered to be multidisciplinary pain centers.

^*∗*^According to 2009 guidelines

^*∗∗*^According to 1990 guidelines.

^*∗∗∗*^No citation provided: unclear what guidelines were used.

**Table 9:** Useful but infrequently reported survey questions.

Variable in questionnaire	Number of articles reporting on it
Average cost of sessions, common treatments	3
Average length of the first appointment	2
Multidisciplinary pain facility ownership	2
Guidelines used	1
Inclusion criteria for referrals	5
Location of multidisciplinary pain facilities (e.g., in hospital, free-standing)	2
Major medical equipment available	1
Measure of data completeness and protocol for dealing with missing data	2
Measures of treatment effectiveness/outcome measures	1
Number of providers at each facility	2
Percentage of facilities that are urban or rural	3
Percentage of facilities that are public or private	4
Percentage of facilities treating children	3
Source of public and/or private funding	3

## Appendices

### A. Search Strategy for MEDLINE (OVID)

(1) Pain/
(2) Pain.mp. [mp = title, abstract, original title, name of substance word, subject heading word, keyword heading word, protocol supplementary concept word, rare disease supplementary concept word, unique identifier]
(3) Pain clinics/
(4) Pain clinic^*∗*^.mp. [mp = title, abstract, original title, name of substance word, subject heading word, keyword heading word, protocol supplementary concept word, rare disease supplementary concept word, unique identifier]
(5) Pain service^*∗*^.mp. [mp = title, abstract, original title, name of substance word, subject heading word, keyword heading word, protocol supplementary concept word, rare disease supplementary concept word, unique identifier]
(6) Pain cent^*∗*^.mp. [mp = title, abstract, original title, name of substance word, subject heading word, keyword heading word, protocol supplementary concept word, rare disease supplementary concept word, unique identifier]
(7) Pain facilit^*∗*^.mp. [mp = title, abstract, original title, name of substance word, subject heading word, keyword heading word, protocol supplementary concept word, rare disease supplementary concept word, unique identifier]
(8) Pain management clinic^*∗*^.mp. [mp = title, abstract, original title, name of substance word, subject heading word, keyword heading word, protocol supplementary concept word, rare disease supplementary concept word, unique identifier]
(9) Pain management service^*∗*^.mp. [mp = title, abstract, original title, name of substance word, subject heading word, keyword heading word, protocol supplementary concept word, rare disease supplementary concept word, unique identifier]
(10) Pain management cent^*∗*^.mp. [mp = title, abstract, original title, name of substance word, subject heading word, keyword heading word, protocol supplementary concept word, rare disease supplementary concept word, unique identifier]
(11) Pain management facility^*∗*^.mp. [mp = title, abstract, original title, name of substance word, subject heading word, keyword heading word, protocol supplementary concept word, rare disease supplementary concept word, unique identifier]
(12) Pain treatment clinic^*∗*^.mp. [mp = title, abstract, original title, name of substance word, subject heading word, keyword heading word, protocol supplementary concept word, rare disease supplementary concept word, unique identifier]
(13) Pain treatment service^*∗*^.mp. [mp = title, abstract, original title, name of substance word, subject heading word, keyword heading word, protocol supplementary concept word, rare disease supplementary concept word, unique identifier]
(14) Pain treatment cent^*∗*^.mp. [mp = title, abstract, original title, name of substance word, subject heading word, keyword heading word, protocol supplementary concept word, rare disease supplementary concept word, unique identifier]
(15) Pain treatment facilit^*∗*^.mp. [mp = title, abstract, original title, name of substance word, subject heading word, keyword heading word, protocol supplementary concept word, rare disease supplementary concept word, unique identifier]
(16) Health surveys/or health care surveys/
(17) Survey^*∗*^.mp. [mp = title, abstract, original title, name of substance word, subject heading word, keyword heading word, protocol supplementary concept word, rare disease supplementary concept word, unique identifier]
(18) Program evaluation/
(19) Program evaluation.mp. [mp = title, abstract, original title, name of substance word, subject heading word, keyword heading word, protocol supplementary concept word, rare disease supplementary concept word, unique identifier]
(20) Questionnaires/
(21) Questionnaire^*∗*^.mp. [mp = title, abstract, original title, name of substance word, subject heading word, keyword heading word, protocol supplementary concept word, rare disease supplementary concept word, unique identifier]
(22) Program assessment.mp. [mp = title, abstract, original title, name of substance word, subject heading word, keyword heading word, protocol supplementary concept word, rare disease supplementary concept word, unique identifier]
(23) Program outcome.mp. [mp = title, abstract, original title, name of substance word, subject heading word, keyword heading word, protocol supplementary concept word, rare disease supplementary concept word, unique identifier]
(24) #1 or #2
(25) #3 or #4 or #5 or #6 or #7 or #8 or #9 or #10 or #11 or #12 or #13 or #14 or #15
(26) #16 or #17 or #18 or #19 or #20 or #21 or #22 or #23
(27) #24 or #25
(28) #26 or #27

### B. Search Strategy for CINAHL and PsycINFO (ProQuest)

ALL (pain) AND ALL (“pain clinic^*∗*^” OR “pain service^*∗*^” OR “pain cent^*∗*^” OR “pain facilit^*∗*^” OR “pain management clinic^*∗*^” OR “pain management service^*∗*^” OR “pain management cent^*∗*^” OR “pain management facilit^*∗*^” OR “pain treatment clinic^*∗*^” OR “pain treatment service^*∗*^” OR “pain treatment cent^*∗*^” OR “pain treatment facilit^*∗*^”) AND ALL (“survey^*∗*^” OR “program evaluation” OR “program assessment” OR “program outcome” OR “questionnaire^*∗*^”).
